# Non-Destructive Testing for Cavity Damages in Automated Machines Based on Acoustic Emission Tomography

**DOI:** 10.3390/s22062201

**Published:** 2022-03-11

**Authors:** Yueyuan Su, Longjun Dong, Zhongwei Pei

**Affiliations:** 1 School of Automation, Central South University, Changsha 410083, China; 8207191421@csu.edu.cn; 2 School of Resources and Safety Engineering, Central South University, Changsha 410083, China; lj.dong@csu.edu.cn

**Keywords:** non-destructive testing, acoustic emission tomography, cavity damage detection, ray tracing

## Abstract

Damage detection is important for the maintenance of automated machines. General non-destructive testing techniques require static equipment and complex analysis processes, which restricts the maintenance of automated machines. Therefore, this paper proposes an acoustic emission (AE) tomography method for detecting cavity damage in automated machines, combining the fast sweeping method (FSM) and the limited-memory Broyden-Fletcher-Goldfarb-Shanno (L-BFGS) method. This approach overcomes the limitations of real-time AE detection for cavity damage in continuous and homogeneous materials. The proposed method has been applied in numerical and laboratory experiments to validate its feasibility. The results show that the inversed low-velocity regions correspond to the actual cavity regions, and the sources of cavity damage can be effectively detected. This paper provides a new perspective for AE testing technologies, and also lays the foundation for other non-destructive testing techniques, in terms of cavity damage detection.

## 1. Introduction

With the development of electrical automation technologies, automated machines have played an important role in engineering. However, automated machines are inevitably affected by damage sources in continuous and mechanized production processes, which brings great challenges to the safety of enterprises. Various damages and defects affect the health condition of equipment components, such as the rolling bearings [1,2], rotating machinery [3], and cylinders [4], and induce huge hazards to enterprises.

The damage detection technologies for automated machines are commonly divided into general testing technologies and non-destructive testing technologies. General testing technologies need to sample research materials and conduct a destructive test, which is not conducive to the maintenance of the automated machine. Non-destructive testing technologies, including magnetic particle testing [5,6,7], radiographic testing [8], and eddy current testing [9], can detect the health condition without damaging the tested materials. However, some common non-destructive testing technologies also face problems such as the requirement to keep the tested equipment static, complex analysis processes, and so on. This greatly restricts the non-stop maintenance of automated machines in engineering. However, compared with other non-destructive testing technologies, acoustic emission (AE) technology can realize real-time monitoring using acoustic signals [10]. In addition, AE tomography can further detect the distribution of damage sources when the variation in the velocity field is caused by the damage sources. This is of great importance for the real-time monitoring of automated machines in engineering.

AE technology is an important and basic non-destructive testing technology that is widely used for the detection of damages and defects [11,12]. Thomas Krause proposed a signal model-based AE detection algorithm, and utilized low-frequency signals to detect damage events in wind turbine rotor blades [13]. Dong [14] utilized AE technology to investigate the qualitative relationship between precursors and principal stress direction, and proposed a new analysis method for stability monitoring in engineering. Zhao [15] investigated the tensile deformation characteristics and damage evolution of aluminum alloy sheets, based on AE technology. The tradition AE methods, such as multiple mode decomposition methods, can be used with AE data to identify a single region of damage on a uniform background. However, the distribution of velocity and damage sources is usually complex in engineering, and the noise is also inevitable to the frequency of the sampled signal. This restricts the application of multiple mode decomposition methods. Additionally, identifying the AE damage location is an important application of AE technology. Dong [16] developed an improved A* search algorithm and realized the high-precision source location under complex three-dimensional structures with irregular holes. Wei [17] proposed a dynamic damage location method for a high-speed train bogie, and accurately detected damage sources through signal reconstruction and localization. Dong [18] investigated the effect of temperature on the location accuracy in AE experiments, and found that the accuracy decreased sharply due to cracks in the heating process. Dong [19] proposed an AE location method, and realized the accurate location in complex structures containing unknown empty areas. However, for metallic engineering structures, the velocity distribution is complex, and can be variable in different directions. The assumption of constant velocity might induce errors in source detection. To overcome this limitation, AE tomography has recently been developed [20,21,22]. Jiang [23] proposed an AE tomography method, based on simultaneous algebraic reconstruction, to visualize the internal damages in a steel plate. Dong [24] proposed a new method, combining the improved A* search algorithm and the match method for empty region identification in complex two-dimensional structures. Nicolas Brantut [25] proposed active–passive acoustic emission tomography to monitor laboratory rock deformation experiments. Most AE tomography research focuses on continuous and homogeneous materials, but ignores the detection of cavity damage sources. Cavity damage sources have a great impact on the mechanical properties of metallic materials in automated machines. Research on the real-time detection of cavity damage in automatic machines, based on AE tomography technology, plays an important role in ensuring the safe operation of equipment.

In this paper, AE tomography, combining the fast sweeping method (FSM) and the limited-memory Broyden-Fletcher-Goldfarb-Shanno (L-BFGS) method, is proposed for detecting cavity damage sources. This method utilizes the optimization of misfit between the observed and calculated arrivals, to literately update the velocity distribution from the background velocity. Numerical and laboratory experiments are conducted to verify the identification performance of the proposed method for cavity damage sources.

## 2. Methods

AE tomography is composed of forward and inversion parts. The forward part is also called forward modeling, which calculates the arrivals of nodes in the research region. The arrivals of nodes are directly related to the velocity distribution of the research region. The arrivals where the receivers are located are picked up in the forward part, and then compared with the observed arrivals in the inversion part. When the misfit between the calculated and observed arrivals is higher than the convergency requirement, an inversion algorithm is used to update the velocity field. This procedure does not stop until it converges or reaches a certain iteration.

### 2.1. Forward Modeling

Under the condition of high-frequency approximation, the wavefront of an elastic wave in an isotropic medium is an approximately satisfied eikonal equation, as follows:

(1) $$\left|\nabla T\left(x\right)\right|=\frac{1}{c\left(x\right)},x\;\in \Omega ,$$

where the boundary condition is $t\left(x_{s}\right)=0$. The fast sweeping method (FSM) calculates arrivals based on the upwind method and the alternating scanning sequence of a Gauss–Seidel iteration [26]. It can provide highly accurate and efficient computation for the forward part, which is important in the tomography process. The FSM is used to solve the eikonal equation in the discrete case. For simplicity, the FSM is introduced in the two-dimensional case. In FSM, the discrete eikonal equation is written as follows:

(2) $${\left[{\left(t_{i,j}-t_{x\;\min}\right)}^{+}\right]}^{2}+{\left[{\left(t_{i,j}-t_{y\;\min}\right)}^{+}\right]}^{2}=\frac{h^{2}}{c_{i,j}}$$

where

(3) $$\{\begin{array}{c} t_{x\;\min}=\min\left(t_{i-1,j},\;\;t_{i+1,j}\right),\\ t_{y\;\min}=\min\left(t_{i,j-1},\;\;t_{i,j+1}\right), \end{array}$$

and

(4) $$x^{+}=\{\begin{array}{c} x,\;\;\;x>0,\\ 0,\;\;\;x\leq 0. \end{array}$$

The unique solution to Equation (2) is as follows:

(5) $$t_{i,j}=\{\begin{array}{cc} \min\left(a,b\right)+\frac{h}{c_{i,j}}, & \left|a-b\right|\geq \frac{h}{c_{i,j}},\\ \frac{a+b+\sqrt{\frac{2h^{2}}{c_{i,j}^{2}}-{\left(a-b\right)}^{2}}}{2}, & \left|a-b\right|<\frac{h}{c_{i,j}}, \end{array}$$

where $a=t_{x\;\min}$ and $b=t_{y\;\min}$. To determine the unique solution, FSM consists of the following three steps:

1. Initialization: initializing the model with $t_{s}=0$, and assigning large positive values at all other grid points;
2. Gauss-Seidel iteration: sweeping the domain by Gauss-Seidel iterations, and selecting the smaller value between the new solution and the original solution;
3. Convergence: repeating step (2) until $||t_{k+1}-t_{k}||\leq \varepsilon$.

### 2.2. Inversion

To invert the wave velocity field by iteration, we tried to minimize the objective function, as follows:

(6) $$L\left(c\right)=\frac{1}{2}\sum_{i=1}^{m}\sum_{j=1}^{n}{\left[t\left(c,r\right)-t^{obs}\left(r\right)\right]}^{2},$$

where t and $t^{obs}$ represent the values of calculated first arrivals and observed first arrivals, respectively. The gradient of the objective function, with respect to the wave velocity, is implicitly nonlinear. Moreover, the adjoint state method [27] is used to calculate the gradient $L^{\prime }\left(c^{k}\right)$. The adjoint state variable λ is the solution of the following:

(7) ∇·λ∇T=0,

with the following boundary condition:

(8) $$n\cdot \lambda \nabla T=T^{obs}-T,$$

where n represents the unit normal vector. Equation (7) can be rewritten as follows:

(9) $$\frac{d}{dx}\left(a\lambda \right)+\frac{d}{dz}\left(b\lambda \right)=0,$$

where $a=\frac{dt\left(x,z\right)}{dx},b=\frac{dt\left(x,z\right)}{dz}$ can be calculated as follows:

(10) $$a_{i-1/2,j}=\frac{t_{i,j}-t_{i-1,j}}{\Delta x},a_{i+1/2,j}=\frac{t_{i+1,j}-t_{i,j}}{\Delta x},$$

(11) $$b_{i,j-1/2}=\frac{t_{i,j}-t_{i,j-1}}{\Delta z},b_{i,j+1/2}=\frac{t_{i,j+1}-t_{i,j}}{\Delta z},$$

Then, the values for $a_{i-1/2,j}^{\pm },\;a_{i+1/2,j}^{\pm },\;b_{i,j-1/2}^{\pm },\;a_{i,j+1/2}^{\pm }$ with itself and its absolute value can be calculated. Take $a_{i-1/2,j}^{\pm }$, for instance, as follows:

(12) $$a_{i-1/2,j}^{+}=\frac{a_{i-1/2,j}+\left|a_{i-1/2,j}\right|}{2},$$

Following this, the adjoint variable is obtained from Equation (9), with a finite difference scheme and gradient, as follows:

(13) $$\tilde{c}=-{\left(I-v\Delta \right)}^{-1}\left(\frac{1}{c^{3}}\sum^{\;}\lambda \right),$$

After determining the adjoint variable and gradient, we adopted the L-BFGS method for numerical optimization. The L-BFGS algorithm can mitigate the computational cost in the inversion part by replacing the approximate Hessian matrix with updated values for the model and its gradient. The L-BFGS algorithm is of importance for realizing fast tomography for non-destructive testing. The iteration of inversion starts with a given initial velocity model $c_{0}$, and the iterative process can be formulated as follows

(14) $$c^{k+1}=c^{k}-\alpha^{k}{\tilde{c}}^{k},$$

where ${\tilde{c}}^{k}$ represents the exploration direction of the model in a single iteration, and $\alpha^{k}$ is the iteration step size, determined by an inaccurate linear search based on Wolfe–Powell conditions. The exploration direction ${\tilde{c}}^{k}$ is estimated by the following:

(15) $${\tilde{c}}^{k}=A_{k}^{-1}L^{\prime }\left(c^{k}\right),$$

where *A_k_* is a positive definite operator satisfying the following condition:

(16) $$A_{k+1}\left(c^{k+1}-c^{k}\right)=L^{\prime }\left(c^{k+1}\right)-L^{\prime }\left(c^{k}\right),$$

L-BFGS is one of the quasi-Newton methods, so it has an approximate second-order convergence rate. As for the quasi-Newton method, the positive definite operator Ak is obtained by iterative calculation to approximately replace the Hessian matrix, which not only has a super linear convergence speed, but also effectively saves the required memory.

When the observations are not accurate enough, or the initial model is far from the real model, the algorithm may not find the correct descent direction, or cannot calculate the iterative step size. Therefore, in each iteration, proper regularization is necessary, which helps keep the algorithm stable.

**Figure 1:**
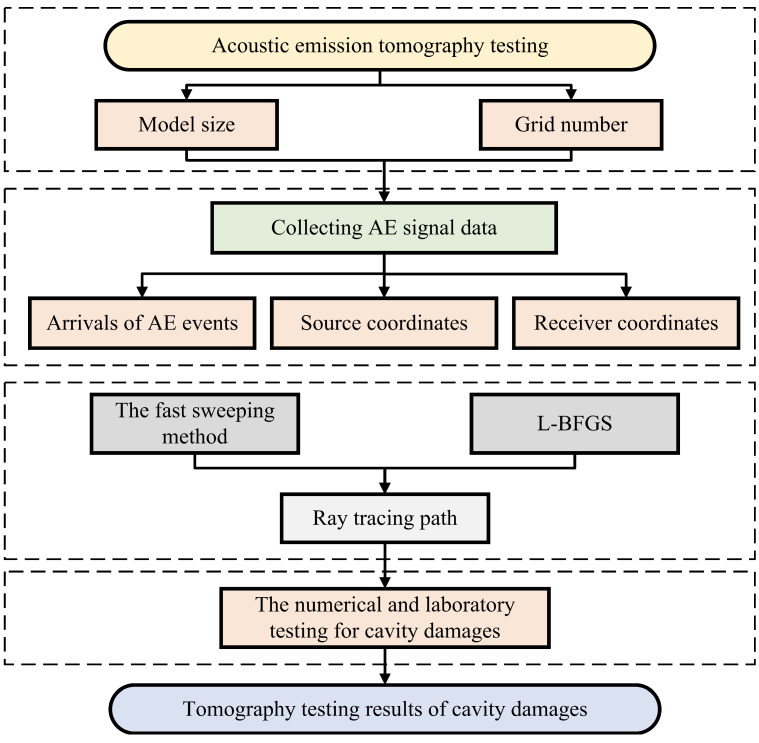
Flowchart of tomography testing for cavity damages. The proposed method utilizes FSM and L-BFGS as the forward modeling and inversion algorithm, respectively.

AE tomography testing consists of the following three steps, and is shown in Figure 1:

1. Establishing the initial model and its grid size according the region of interest;
2. Collecting AE signal data, such as arrivals of AE events, source and receiver coordinates;
3. Calculating the inversion result from the initial model, based on FSM and L-BFGS, until it satisfies the convergence requirement. The convergence requirement is set so that when the difference of the updated value of the model is smaller than a certain value, or the iteration reaches a certain value, the inversion process is regarded as satisfying the convergence.

## 3. Experiments

To verify the feasibility of the proposed method for detecting cavity damage sources, we conducted numerical and laboratory experiments, which are described in this section. The numerical experiments were assumed for a 40 cm×40 cm two-dimensional plane, where the background velocity was set as 3000 m/s. Forty sensors were evenly arranged on the two sides, and the sensors on the top side were used as the AE sources. Four hundred AE signals were received by the sensors on the bottom side. The coordinates of the sensors were known, and each AE event could be received by the sensors. In the first numerical experiment, there was a single cavity damage source, whose size was 15 cm×15 cm. The second numerical experiment contained multiple cavity damage sources, each 7.5 cm×7.5 cm in size. The numerical experiments were conducted to verify the performance of the proposed method for detecting cavity damage sources in an ideal situation. The laboratory experiment was conducted with a 40 cm×40 cm two-dimensional steel plate containing a 16 cm×10 cm cavity damage source. There were sixteen sensors arranged on the two opposite sides, and sixty-four arrivals were received and used to reconstruct the velocity field from the initial model.

### 3.1. Numerical Experiments

**Figure 2:**
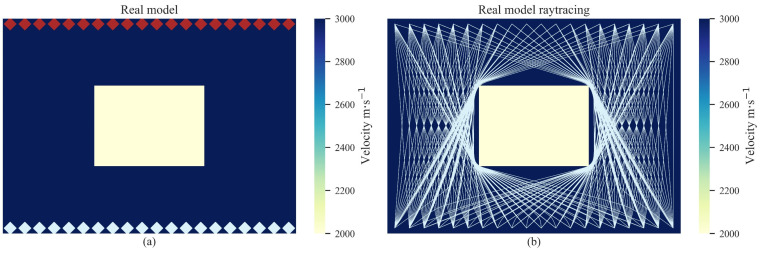
The real model (**a**) and its ray-tracing path (**b**) in the numerical experiments for the single cavity damage source. The red rhombuses represent the AE signal sources and the white rhombuses represent the receivers.

**Figure 3:**
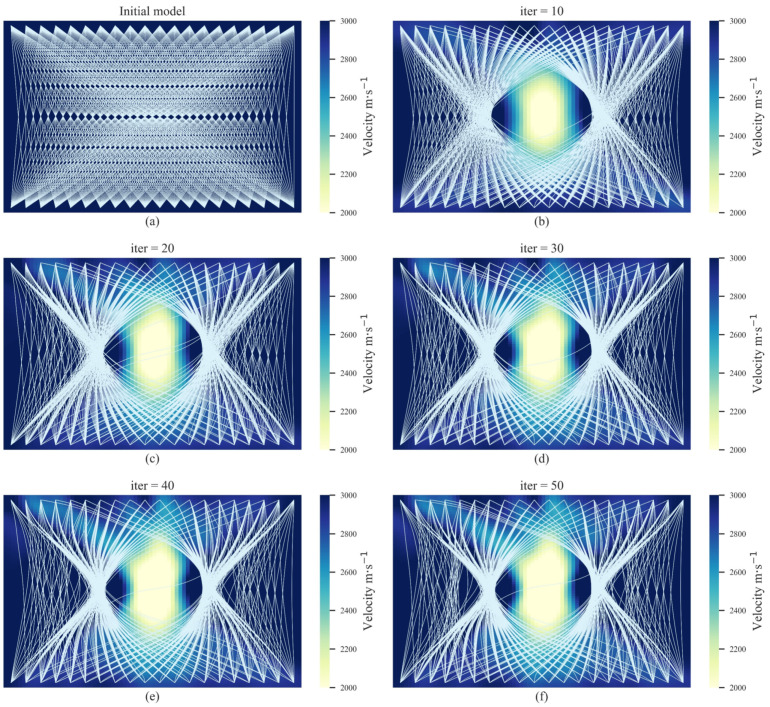
The iteration results for AE tomography in the numerical experiments for the single cavity damage source. (**a**–**f**) show the iteration results from the initial model to the 50th iteration.

To validate the AE tomography method for detecting cavity damage sources under theoretical conditions, the first numerical experiment and its ray-tracing paths are shown in Figure 2. It can be observed from Figure 2 that the red rhombuses represent the AE signal sources and the white rhombuses represent the receivers. The background velocity was set as *V* = 3000 m/s. Since the velocity in the cavity damage region is lower than the background velocity, the ray-tracing paths tend to pass around the cavity damage region. This apparent diffraction phenomenon is shown in Figure 2b. The background velocity was set as the initial model for the iteration, and the iteration results are shown in Figure 3. It can be observed that, with an increasing number of iterations, a low-velocity region gradually appears in the inversion region. The velocity difference between the inversion region and the actual cavity damage region demonstrates a significant uptrend. In addition, the ray-tracing paths in the inversion regions also tend to bend and diffract with the increase in iterations. This is because the actual cavity damage region limits the velocity of the first arrival wave through the receivers on the bottom sides, and the first arrival wave has to travel around the boundaries to the receivers. Thus, the arrivals of the receivers in the real model are generally slower than those in the initial model. The difference generally increases with the difference in velocity between the real and initial models, and then remains unchanged. This is because when the velocity in the damaged regions is lower than a certain value, rays do not passed, and, as a result, the variation in velocity will not further increase the difference. Since the optimization of tomography can be regarded as a solution to an ill-posed equation, the amount of available data is always less than the velocity cells of the model. The difference between the obtained and actual low-velocity regions can only be minimized when the difference is large enough to affect the received arrivals. Thus, the obtained results might be relatively different compared with the actual low-velocity regions. The final tomography result in Figure 3 shows that the proposed method can realize the reconstruction of a single cavity damage source in the inversion region.

**Figure 4:**
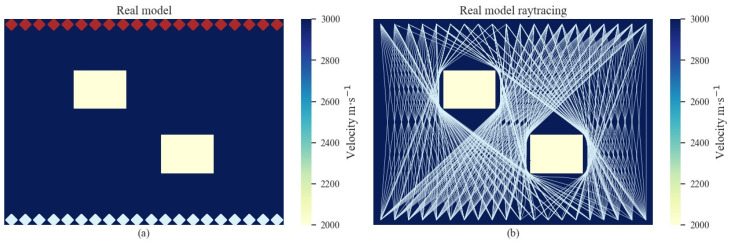
The real model (**a**) and its ray-tracing path (**b**) in the numerical experiments for multiple cavities. The red rhombuses represent the AE signal sources and the white rhombuses represent the receivers.

**Figure 5:**
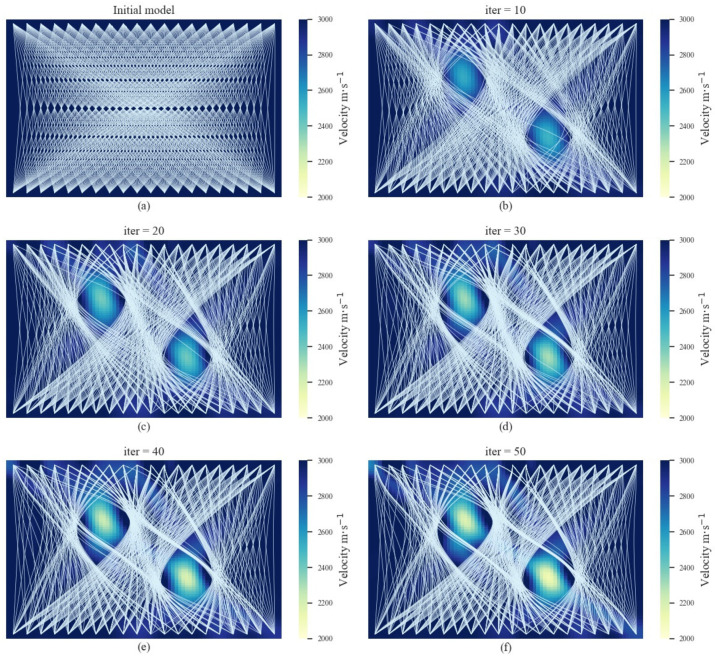
The iteration results of AE tomography in the numerical experiments for multiple cavity damage sources. (**a**–**f**) show the iteration results from the initial model to the 50th iteration.

The second numerical experiment and its ray-tracing paths are shown in Figure 4. In contrast to the first numerical experiment, the real model contains two cavity damage sources, and its ray-tracing paths are more complex. The cavity damage sources block the propagation of signals between the left diagonal sensors. The background velocity was set as *V* = 3000 m/s for the initial model, and the tomography results are shown in Figure 5. There is no diffraction in the initial model, and the rays travel directly from the sources to the receivers at iter. = 0. As the iteration number increases, the low-velocity regions become apparent, where the rays travel around the damage regions. The sizes of the low-velocity regions become larger with each iteration. It can be observed from Figure 5f that the low-velocity regions correspond to the actual cavity damage sources.

The numerical experiments demonstrated the feasibility of the proposed AE tomography method for detecting cavity damage sources from the initial model. The inversed low-velocity regions were located at the position of actual cavity damage sources, surrounded by the diffracting ray-tracing paths.

### 3.2. Laboratory Experiments

Compared to theoretical numerical experiments, there are unavoidable errors and factors in laboratory AE tomography experiments, such as the uniform real velocity distribution, arrival errors, and so on. Therefore, it was necessary to validate the performance of the proposed method in the laboratory.

**Figure 6:**
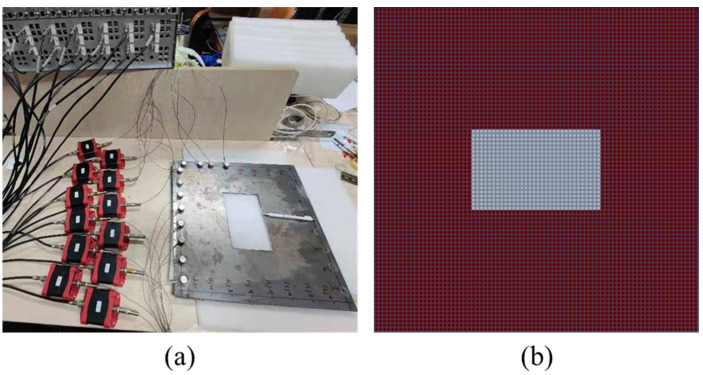
Laboratory AE tomography experiments: (**a**) experimental platform for detecting cavity damage; (**b**) mesh grids for the experiment, white grids represent cavity damage regions and red grids represent the steel plate.

The experimental platform for detecting cavity damage is shown in Figure 6a. The inversion region was a steel plate with a cavity region. The sensors were arranged on the steel plate, and the received wave belonged to the surface wave. The AE signals were activated by the pulse transmitted function of the active AE source, and were detected by the receivers. Figure 6b shows the 80×80 mesh grids for the laboratory experiments. The red mesh grid represents the steel plate and the white mesh grid represents the cavity region.

**Figure 7:**
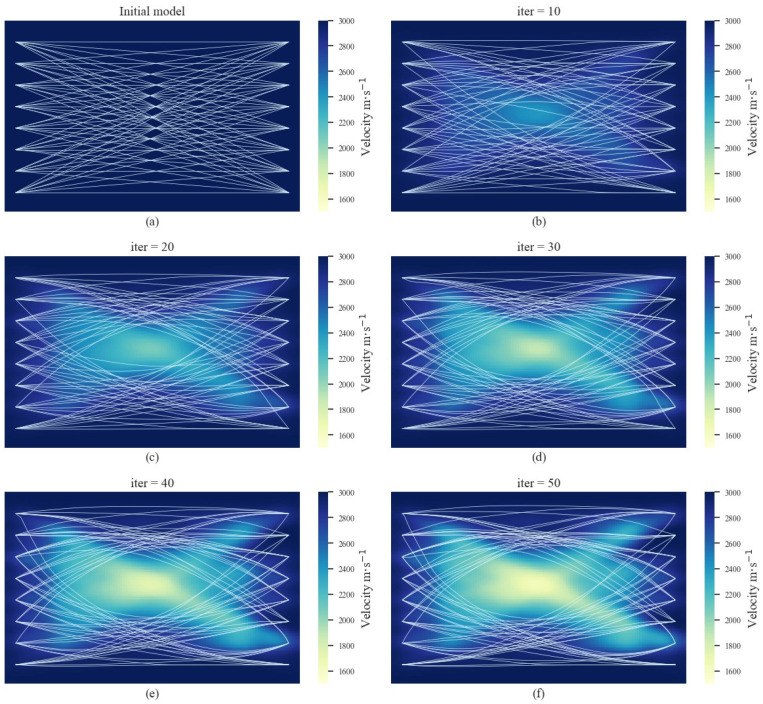
The iteration results of AE tomography in the laboratory experiments. (**a**–**f**) show the iteration results from the initial model to the 50th iteration.

The results of AE tomography in the laboratory experiments are shown in Figure 7. The initial model was established based on the average velocity obtained from the signals excited and received by sensors on the same side. The initial model was set with a background velocity of V=3000 m/s. As the iterations increased, it can be observed that the velocity of the middle region begins to decrease, and the diffraction of rays increased. When the iterations reached 40, there was an apparent rectangular low-velocity region, corresponding to the cavity region. Due to the impact of error factors, there were artifacts along the direction between the sensors. This was mainly because the velocity gradient along the direction between the sensors was often higher than other areas, and the velocity tended to change. inevitably. In addition, cutting the steel plate could have caused invisible damage to the uncut parts around it. Although no cracks were observed on the surface, the internal wave velocity field had changed. While the results were affected by these artifacts, to some extent, the actual distribution of the cavity region could still be clearly identified. This proves the feasibility of the proposed AE tomography for detecting cavity damage sources in automated machines.

## 4. Conclusions

Acoustic emission tomography is a new kind of non-destructive testing technology. This paper proposes an AE tomography method for detecting cavity damage sources in automated machines. It utilizes FSM as the forward modeling method, to calculate the arrivals in the inversion region. Subsequently, the misfit function between the observed and calculated arrivals is iteratively minimized with an optimization algorithm, based on the L-BFGS method. To validate the feasibility of the proposed method for detecting cavity damage, numerical and laboratory experiments were conducted. The reliability and feasibility of the proposed method were proved by the tomography results. The proposed method can realize the detection of cavity damage in theoretical and laboratory situations. The position of the low-velocity region accurately indicates the actual cavity damage sources. Therefore, the proposed method satisfies the requirement of accuracy for the detection of cavity damage sources in automated machines.

The proposed method was verified by the detection of cavity damage sources in the paper, but it may be suitable for other damage sources, such as cracks and corrosion. This is because the proposed method is based on the variation in wave velocity after the damage. The proposed AE tomography method offers promising insights into damage detection in automated machines, based on non-destructive testing technology. This study not only provides a theoretical and technical basis for the application of this technology in steel-made equipment, but also it could be extended to detect damage in other composite materials.

## Data Availability

Not applicable.

## References

[1] Cerrada M., Sánchez R.-V., Li C., Pacheco F., Cabrera D., Valente de Oliveira J., Vásquez R.E. (2018). A review on data-driven fault severity assessment in rolling bearings. Mech. Syst. Signal Process..

[2] Goyal D., Choudhary A., Pabla B.S., Dhami S.S. (2020). Support vector machines based non-contact fault diagnosis system for bearings. J. Intell. Manuf..

[3] Lei Y., Lin J., He Z., Zuo M.J. (2013). A review on empirical mode decomposition in fault diagnosis of rotating machinery. Mech. Syst. Signal Process..

[4] Hamzeloo S.R., Shamshirsaz M., Rezaei S.M. (2012). Damage detection on hollow cylinders by Electro-Mechanical Impedance method: Experiments and Finite Element Modeling. Comptes Rendus Mécanique.

[5] Zolfaghari A., Zolfaghari A., Kolahan F. (2018). Reliability and sensitivity of magnetic particle nondestructive testing in detecting the surface cracks of welded components. Nondestruct. Test. Eval..

[6] Tout K., Meguenani A., Urban J.-P., Cudel C. (2021). Automated vision system for magnetic particle inspection of crankshafts using convolutional neural networks. Int. J. Adv. Manuf. Technol..

[7] Zhang M., Zhang X., Li M., Cao J., Huang Z. (2020). Optimization Design and Flexible Detection Method of a Surface Adaptation Wall-Climbing Robot with Multisensor Integration for Petrochemical Tanks. Sensors.

[8] Wang X., Wong B.S., Tan C., Tui C.G. (2011). Automated Crack Detection for Digital Radiography Aircraft Wing Inspection. Res. Nondestruct. Eval..

[9] Rifai D., Abdalla A., Ali K., Razali R. (2016). Giant Magnetoresistance Sensors: A Review on Structures and Non-Destructive Eddy Current Testing Applications. Sensors.

[10] Dong L., Zou W., Li X., Shu W., Wang Z. (2019). Collaborative localization method using analytical and iterative solutions for microseismic/acoustic emission sources in the rockmass structure for underground mining. Eng. Fract. Mech..

[11] Dong L., Zhang Y., Ma J. (2020). Micro-Crack Mechanism in the Fracture Evolution of Saturated Granite and Enlightenment to the Precursors of Instability. Sensors.

[12] Ma J., Dong L., Zhao G., Li X. (2019). Focal Mechanism of Mining-Induced Seismicity in Fault Zones: A Case Study of Yongshaba Mine in China. Rock Mech. Rock Eng..

[13] Krause T., Ostermann J. (2020). Damage detection for wind turbine rotor blades using airborne sound. Struct. Control Health Monit..

[14] Dong L., Chen Y., Sun D., Zhang Y. (2021). Implications for rock instability precursors and principal stress direction from rock acoustic experiments. Int. J. Min. Sci. Technol..

[15] Zhao P., Sun Y., Jiao J., Fang G. (2020). Correlation between acoustic emission detection and microstructural characterization for damage evolution. Eng. Fract. Mech..

[16] Dong L., Hu Q., Tong X., Liu Y. (2020). Velocity-Free MS/AE Source Location Method for Three-Dimensional Hole-Containing Structures. Engineering.

[17] Wei X., Chen Y., Lu C., Chen G., Huang L., Li Q. (2020). Acoustic emission source localization method for high-speed train bogie. Multimed. Tools Appl..

[18] Dong L., Tao Q., Hu Q. (2021). Influence of temperature on acoustic emission source location accuracy in underground structure. Trans. Nonferrous Met. Soc. China.

[19] Dong L., Tao Q., Hu Q., Deng S., Chen Y., Luo Q., Zhang X. (2022). Acoustic emission source location method and experimental verification for structures containing unknown empty areas. Int. J. Min. Sci. Technol..

[20] Pei N., Shang J., Bond L.J. (2021). Analysis of Progressive Tensile Damage of Multi-walled Carbon Nanotube Reinforced Carbon Fiber Composites by Using Acoustic Emission and Micro-CT. J. Nondestruct. Eval..

[21] Yang J., Mu Z.-L., Yang S.-Q. (2020). Experimental study of acoustic emission multi-parameter information characterizing rock crack development. Eng. Fract. Mech..

[22] Al-Jumaili S.K., Eaton M.J., Holford K.M., Pearson M.R., Crivelli D., Pullin R. (2018). Characterisation of fatigue damage in composites using an Acoustic Emission Parameter Correction Technique. Compos. Part B Eng..

[23] Jiang Y., Xu F., Xu B. (2015). Acoustic Emission tomography based on simultaneous algebraic reconstruction technique to visualize the damage source location in Q235B steel plate. Mech. Syst. Signal Process..

[24] Dong L., Tong X., Hu Q., Tao Q. (2021). Empty region identification method and experimental verification for the two-dimensional complex structure. Int. J. Rock Mech. Min. Sci..

[25] Brantut N. (2018). Time-resolved tomography using acoustic emissions in the laboratory, and application to sandstone compaction. Geophys. J. Int..

[26] Zhao H. (2004). A fast sweeping method for Eikonal equations. Math. Comput..

[27] Leung S., Qian J. (2006). An adjoint state method for three-dimensional transmission traveltime tomography using first-arrivals. Commun. Math. Sci..

